# Genetic correction of splice site mutation in purified and enriched myoblasts isolated from mdx^5cv ^mice

**DOI:** 10.1186/1471-2199-10-15

**Published:** 2009-02-23

**Authors:** Katie Maguire, Takayuki Suzuki, Darlise DiMatteo, Hetal Parekh-Olmedo, Eric Kmiec

**Affiliations:** 1 Department of Biological Sciences, University of Delaware, Newark, DE 19716, USA; 2 Department of Neurology and Neurological Sciences, Stanford University School of Medicine, Stanford, CA 94305, USA

## Abstract

**Background:**

Duchenne Muscular Dystrophy (DMD) is an X-linked genetic disorder that results in the production of a dysfunctional form of the protein, dystrophin. The mdx^5cv ^mouse is a model of DMD in which a point mutation in exon 10 of the dystrophin gene creates an artificial splice site. As a result, a 53 base pair deletion of exon 10 occurs with a coincident creation of a frameshift and a premature stop codon. Using primary myoblasts from mdx^5cv ^mice, single-stranded DNA oligonucleotides were designed to correct this DNA mutation.

**Results:**

Single-stranded DNA oligonucleotides that were designed to repair this splice site mutation corrected the mutation in the gene and restored expression of wild-type dystrophin. This repair was validated at the DNA, RNA and protein level. We also report that the frequency of genetic repair of the mdx mutation can be enhanced if RNAi is used to suppress expression of the recombinase inhibitor protein Msh2 in cultures containing myoblasts but not in those heavily enriched in myoblasts.

**Conclusion:**

Exogenous manipulations, such as RNAi, are certainly feasible and possibly required to increase the successful application of gene repair in some primary or progenitor muscle cells.

## Background

Duchenne Muscular Dystrophy (DMD) is an X-linked recessive disorder with an incidence of 1 in 3,500 live born males. It is caused by the dysfunction of dystrophin (*DMD*), a large 427 kDa cytoplasmic protein that forms a link between actin filaments and the dystrophin-glycoprotein (DGC) complex [1]. The loss of dystrophin is thought to affect the integrity of the cell membrane leading to a disruption in cellular calcium homeostasis and eventually cell necrosis [1-3]. At the genetic level, one-third of boys afflicted with DMD have point mutations, insertions or deletions while the remaining two-thirds have gross deletions or rearrangements within the 2.5 Mb gene [4] [OMIM #310200 Muscular Dystrophy, Dystrophy Type, DMD] and all generally cause a translational frameshift.

Therapeutic strategies such as gene replacement have been used to restore protein expression by effectively re-engineering dystrophin cDNA. In addition, attempts have been made to introduce smaller functional homologs of DMD to cells in an attempt to reverse the muscle degradation process [5-8]. Vectors, which carry these constructs, however, often elicit an immune response [8,9]. An RNA/DNA chimeric oligonucleotide (chimera) has been used successfully to correct the splice site mutation to generate a genetic alteration in the dystrophin gene, in both *ex vivo *and *in vivo *studies in mouse and dog models of DMD [10-13]. In a pioneering study, Bartlett *et al*. [10] showed the correction of the canine dystrophin gene within skeletal muscle fibers after injecting the chimera into the cranial tibialis of Golden Retrievers. The production of wild-type dystrophin was restored and could be detected for up to 48 weeks at the site of the injection. Rando *et al*. [11] demonstrated chimera-directed repair of the dystrophin gene in the mouse extending the results from the dog model; once again the corrected cells were again localized to the injection site.

Antisense oligonucleotides also have been used to alter the splicing of the dystrophin message in a process generally termed, exon skipping [14-16]. A large number of exciting reports have demonstrated the feasibility of altering the splicing patterns of the mutant DMD transcript [15,17-21]. While this approach is likely to have significant therapeutic benefit, the original DMD mutation(s) remains and thus a continuum of antisense ODN delivery will be required [22].

When muscle fibers are damaged within the body, a population of quiescent precursor muscle cells, or satellite cells, become activated myoblasts [23,24], rapidly divide and initiate the regeneration of the injured muscle [24]. In the case of DMD, the muscle is thought to be constantly going through cycles of degeneration and regeneration due to the instability of the musculature and, therefore, abundantly populated with myogenic precursor cells. Thus, an important long term therapeutic approach then would be to isolate and genetically correct the myoblasts at the DNA level with subsequent transplantation to the animal model in order to restore functional dystrophin to the muscle fibers.

We have begun to develop a more defined *ex vivo *strategy in which modified single-stranded oligonucleotides are designed to repair the mutant dystrophin (*ex vivo*) in cell cultures enriched in myoblasts. A current challenge is to establish a frequency of repair that leads to a level of dystrophin expression that can be detected in targeted myoblasts. Previously, Bertoni *et al*. [25] have used fluorescently labeled oligonucleotides to correct a point mutation in cells derived from the dystrophic mdx^5cv ^mice. We now repeat the correction of Dmd point mutation using *unlabeled *modified single stranded oligonucleotides and establish novel quantitative assays to measure the dystrophin production in myoblasts. We also stimulate the correction reaction by using RNAi against Msh2, a known inhibitor of the gene repair reaction [26] and observe higher levels of gene repair.

## Results

### Isolation of primary cultures from dystrophic mdx^5cv ^mice containing myoblasts with determination of isolation efficiency

In the natural muscle environment, satellite cells rest in a quiescent state along the periphery of an intact muscle fiber. When muscle fibers are damaged, muscle precursor cells, referred to as myoblasts, divide and fuse with the damaged muscle fibers to repair them [see [27] for review]. In order to isolate the muscle precursor cells for gene repair experiments, muscle tissue was removed from the limbs of 1 to 3 days old mdx^5cv ^mice, enzymatically digested and plated. A pre-plating technique was used to purify these cells from fibroblasts present in the explant [27,28]. It is known that the fibroblasts adhere to the plate rapidly, usually within 2 hours, and, therefore, the pre-plating technique is used them from the culture by passaging the supernatant at a 2 hour time point (PP1) and again at a 24 hour time point (PP2) [28]. Thus, the supernatant is passaged until PP4 at which time cells are harvested to determine the efficiency of myoblast isolation by incubation with an antibody against desmin (expressed in myoblasts). We estimate the efficiency of myoblast isolation in this experiment to be 53% as determined by counting the number of desmin positive cells and dividing this number by the number of total cells observed in multiple fields of view (data not shown). The remaining 47% are most likely fibroblasts. Thus, in the experiments outlined below, we utilize cultures that contain approximately 50% myoblasts and 50% fibroblasts. Primarily myoblasts are a target because only corrected myoblasts that fuse to form myotubes will be capable of expressing corrected dystrophin.

**Figure 1:**
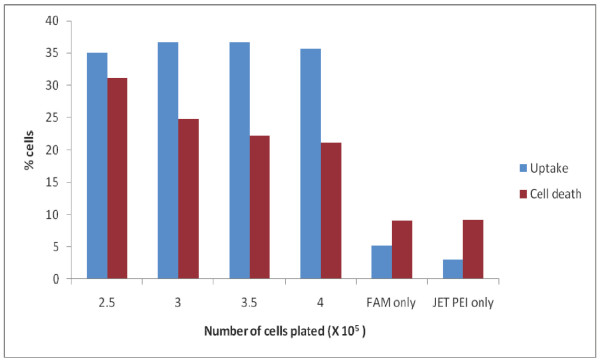
**Optimization of ODN uptake in primary myoblast cultures from mdx^5cv ^mice**. The percent of FAM labeled ODN uptake complexed with the transfection reagent jetPEI (blue bars) as a function of the seeding density of the myoblasts is shown as well as the amount of background fluorescence when myoblasts are incubated with either the FAM oligo or jetPEI alone. The cell density is the 2.5 × 10^6 ^when the cells are treated with FAM or jetPEI alone. The amount of cell death at each cell density and control samples is also shown (red bars).

In order to determine the efficiency of gene repair of the chromosomal dystrophin gene in myoblasts, it was important to determine the level of transfection of oligonucleotides. Oligonucleotides in varying amounts were complexed with Fugene 6 to observe the levels of uptake within the myoblasts. Fugene mediated transfection only showed a 4–6% uptake of the FAM-labeled oligonucleotides (data not shown). Therefore, we chose to use jetPEI, which is formulated specifically for delivery of oligonucleotides to deliver the ODN into the primary cultures. Myoblast cultures were seeded at increasing densities and lipoplexes composed of 6 μL of jetPEI and 3 μg of ODN were added to cultures with readout of uptake by FACS analysis after 24 hours. There was no difference in the amount of oligonucleotide uptake at different cell numbers as shown in Figure 1 (average uptake, 36%). FACS was used to quantify uptake of FAM-labeled oligonucleotide as well as cell death by propidium iodide. Thus, a range of cell densities can be transfected using the jetPEI complex.

### Gene repair of dystrophin directed by modified single-stranded oligonucleotides in purified myoblasts from mdx^5cv ^mice

**Figure 2:**
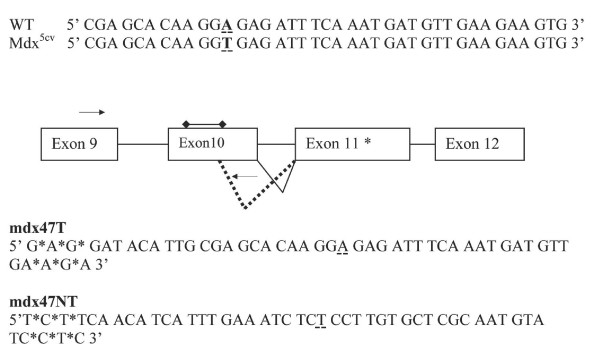
**Mutation in dystrophin gene of mdx^5cv ^mice and correcting ODNs**. Mdx^5cv ^mice have a single point mutation (A to T) in the dystrophin gene, which is both bolded and underlined in the gene sequence above. This single base mutation will create a donor splice site within exon 10 and cause a 53 base portion of this exon to be spliced out of the mature transcript as depicted by the dashed line in the diagram. A frameshift results in a stop codon in exon 11 (*) and will ultimately result in the production of a truncated dystrophin protein. Because the natural donor site is still present in the transcript there is a possibility that some full-length message, containing exon 10 is made and this is shown as the solid line. The ODNs, mdx47T and mdx47NT, shown above, were designed to correct the substitution mutation and the target base is underlined.

The mdx^5cv ^mouse has a point mutation in exon 10, which creates a splice donor site that results in a 53 base deletion within the exon (see diagram in Figure 2). This deletion results in a reading frameshift wherein a premature stop codon is created 95 bases downstream of the mutation site. If the ODN directs correction of the base, then the correct splice pattern is recreated and the 53 deleted bases remain in the message; in effect, restoring the reading frame. To evaluate the restoration of the 53 base pair insertion, a reverse primer was designed to bind to the deleted portion of exon 10. Thus, amplification can only occur if the targeted splice site corrected and the full-length mature message is produced at detectable levels. To determine the level of expression of dystrophin mRNA where the deleted region had been restored, mRNA was reverse-transcribed using a primer specific for exon 12, which is present in all of the transcripts, corrected or not.

We outlined oligonucleotide-mediated gene repair reactions containing ODNs, mdx47NT or mdx47T, designed to correct the T substitution mutation within exon 10 of the dystrophin gene. These are depicted in Figure 2. For clarity, we provide the wild type and mutant DNA sequence, as well. The "T" indicates that the 47-mer is designed to target the transcribed strand while the "NT" refers to a molecule designed to target the non-transcribed strand.

**Figure 3:**
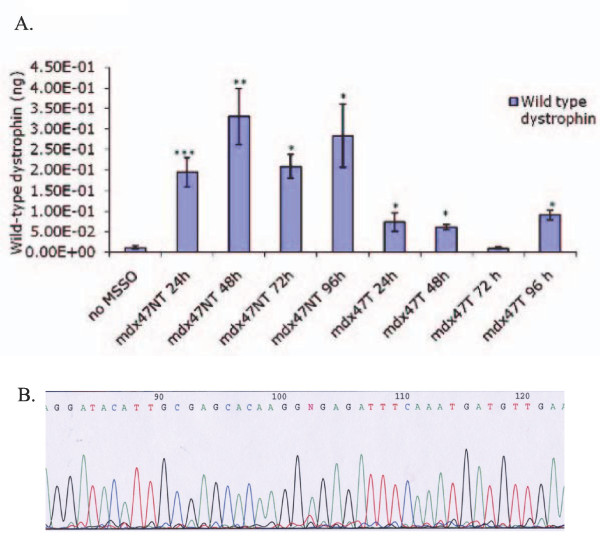
**Quantitative measurement of dystrophin correction in primary cultures containing mdx^5cv ^myoblasts at the DNA level and sequence analysis**. A: Total genomic DNA was isolated from targeted myoblasts at various time points, 24 to 96 hours after introduction of ODNs, mdx47NT or mdx47T. Twenty-five nanograms of this DNA was subjected to absolute quantification by PCR analysis with a FAM labeled probe specific only for the wild-type dystrophin sequence. A standard curve had been previously generated using C2C12 (WT) genomic DNA and the amount of wild-type dystrophin has been extrapolated from this standard and is presented as nanograms of wild-type dystrophin. These data represent the average of three experiments that were analyzed in triplicate. Significance was measured by a Student's t-test and samples were considered significantly different with a *p *value of less than *0.05, **0.01, ***0.001. B: DNA sequence reaction of PCR products showing the chromatogram representing a mixed population at the targeted base (base 102) with adenine (green/corrected base) being the major peak and with thymine (red/mutant base) being a minor population within the reaction.

The oligos were delivered to myoblasts by jetPEI and the cells were allowed to recover for 24, 48, 72 and 96 hours respectively at which point genomic DNA was extracted. Twenty-five nanograms of DNA were subjected to absolute quantification using the Taqman probe analysis. This assay measures the amount of wild-type or corrected dystrophin DNA within each sample. The probe is designed to recognize only dystrophin sequences that contain the changed base and, thus, no direct sequencing of the gene is necessary to confirm sequence alteration [29]. Figure 3A shows that wild-type dystrophin can be detected by 24 hours after transfection with either mdx47NT or mdx47T. The highest amount, 331 pg, a significant 15.72 fold increase (p < 0.001), is detected when cells are targeted with mdx47NT. mdx47T corrects less efficiently with an overall average of 59 pg of wild-type dystrophin detected from 24 to 96 hours, indicating that there is a strand bias toward the nontranscribed strand of the dystrophin gene (Figure 3). PCR products directly from the probe assay were sequenced to confirm the correction within genomic DNA and the sequencing results show that the corrected base (A:red peak) is the major product in the reaction with a very minor peak (T:green peak) being the uncorrected mutant base. This minor mutant peak correlates to the specificity of the probe reaction in that a low level of the mutant base is detected, but as shown in Figure 3A, this background is not statistically significant in the quantitation assay.

To evaluate gene repair at the RNA level, quantitative RT-PCR was carried out using a primer set specific for exon 9 and the deleted portion of exon 10. Table 1 illustrates the cycle number at which the restored portion of the exon initially appears. In these analyses, *higher *Ct values are equivalent to a *lower *amount of corrected message while a lower in Ct values signifies an *increase *in the presence of the deleted portion of exon 10. Table 1 shows that the deleted region is detected at 72 hours of targeting followed by 96 hours in DM. A statistically significant *6-fold increase *in message is detected above nonspecific levels. Taken together, the data indicate that the 47NT is capable of correcting the dystrophin gene and restoring dystrophin expression to the targeted population. Interestingly, the fold increase in correction detected of the dystrophin gene detected with the Taqman probe (15.72×) (see Figure 3A) is not equivalent to the fold increase in expression (6 fold). A likely explanation for this observation indicates that some correction is occurring in fibroblasts, which do not express dystrophin, although such a comparison may not be possible.

**Table 1:** Quantitative measurement of dystrophin, GAPDH, and CKM.

		Recovery Time	
mRNA	Transfected ODN	48 hours	72 hours
		(Ct value)	
Dystrophin	mdx47NT	34.1 ± 0.15	33.8 ± 0.22
	NS	34.9 ± 0.08	37.1 ± 0.35
GAPDH	mdx47NT	13.2 ± 0.08	13.6 ± 0.02
	NS	13.3 ± 0.05	13.6 ± 0.32
CKM	mdx47NT	22.1 ± 0.11	13.6 ± 0.11
	NS	21.5 ± 0.08	13.6 ± 0.10

A quantitative measurement of dystrophin transcripts containing the deleted portion of exon 10 was also performed. The cells were induced to differentiate with addition of media containing 2% horse serum at the indicated time points for 96 hours. Each sample was subjected to RT PCR using either dystrophin specific primers or oligo dT primers. QPCR was performed using a reverse primer specific for the deleted portion of exon 10. GAPDH was used as a loading control and CKM levels were measured to ensure similarities in the extent of differentiation between samples. Significance was measured by a Student's t-test and samples were considered significantly different with a *p *value of less than *0.05, **0.01, ***0.001. mdx47NT was used to correct the mutation while nonspecific ODN was used as a negative control. GAPDH and CKM were used as positive controls with the ODNs in parenthesis added to the mRNA mixtures.

**Figure 4:**
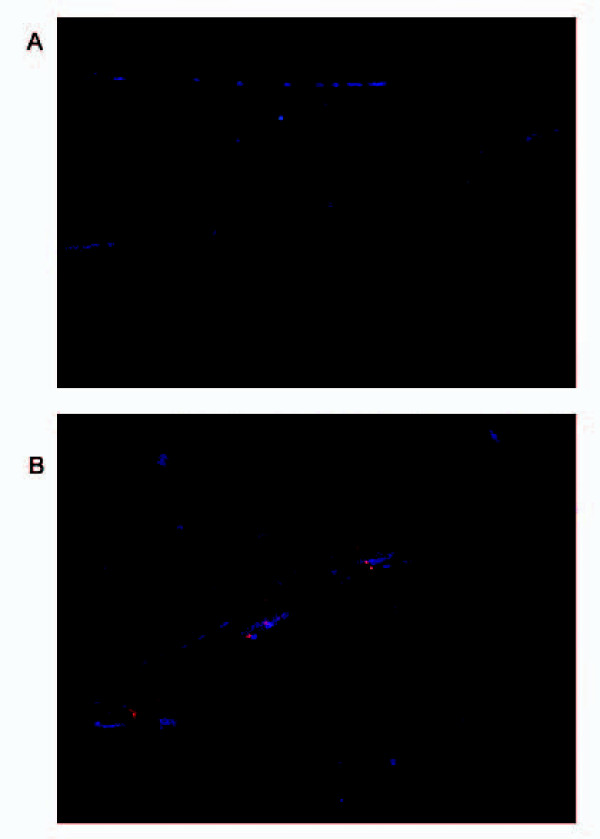
**Detection of dystrophin with exon specific antibodies**. Cultures containing dystrophic myoblasts were seeded in 8 well chambers and were targeted with a 72 hour recovery time after the addition of the either the nonspecific ODN (A) or mdx47NT (B). These cells were then allowed to differentiate for 96 hours at which point they were fixed and incubated with antibody specific for a rabbit polyclonal antibody that recognizes dystrophin and a secondary Alexa fluor labeled antibody (red). The cell nuclei were stained with DAPI, which is shown in blue.

The donor splice site in the DMD gene in mdx^5cv ^mice causes a frameshift which results in a translation of truncated dystrophin protein (10 of the 79 exons). As shown above, correction of the mutation at the DNA level resulted in the restoration of intact dystrophin message. To determine whether the corrected mRNA is translated into normal dystrophin that contains exon 11, cells were seeded and once again transfected with mdx47NT or mdx47T under conditions that provided the highest level of gene correction. The entire assay was performed in microscope chambers so that the samples could be probed directly with an antibody specific for the dystrophin protein. Figure 4 reveals the presence of dystrophin after a 72 hour recovery and 96 hour differentiation period, indicating dystrophin is now being produced within myotubes. Within myotubes that stain positive for dystrophin, it is evident that the protein is distributed throughout (see Figure 4B) These data are in agreement with the western blot analyses performed previously by Bertoni *et al*. [25]. Altogether, the data from DNA, mRNA and protein analyses show that single stranded oligonucleotides without conjugated chemical moieties at their termini (i.e. Cy3) correct the dystrophin gene in cultures containing an enriched population of myoblasts isolated from dystrophic mice.

### Enhancement of dystrophin correction by knockdown of Msh2

**Figure 5:**
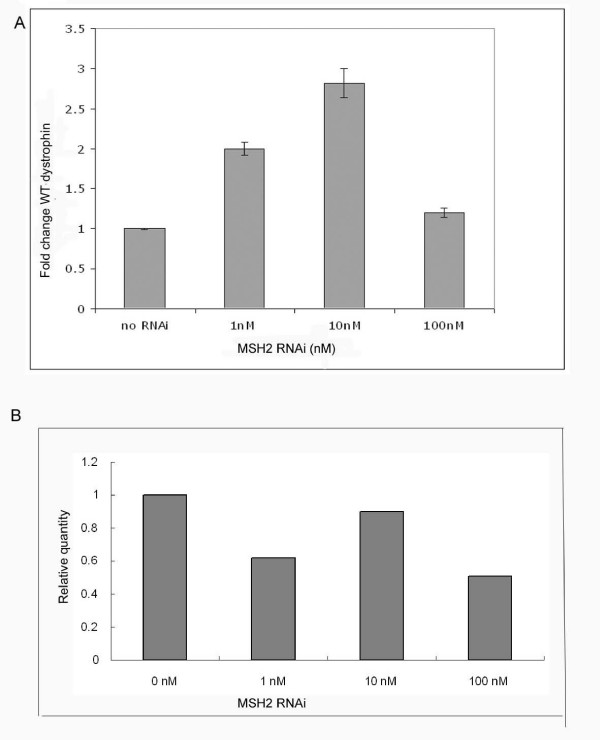
**Enhancement of Dystrophin repair by knockdown of Msh2**. A: Myoblasts derived from mdx^5cv ^mice were co-transfected with various amounts of RNAi specific to knockdown Msh2 and 3 μg of correcting oligonucleotide mdx47NT. Total genomic DNA was isolated after 24 hours and 25 ng were subjected to absolute quantification using a FAM labeled probe specific only for wild type dystrophin. The fold change in wild-type dystrophin was determined from three independent experiments assayed in triplicate and significance was deciphered by a student's t-test. Differences were considered significant only when the p < 0.05. B: Knockdown of Msh2 by RNAi. Myoblasts were co-transfected with various amounts of RNAi specific to knockdown Msh2 and 3 μg of correcting oligonucleotide mdx47NT. Total mRNA was isolated after 6 hours and subjected to RT-PCR.

**Figure 6:**
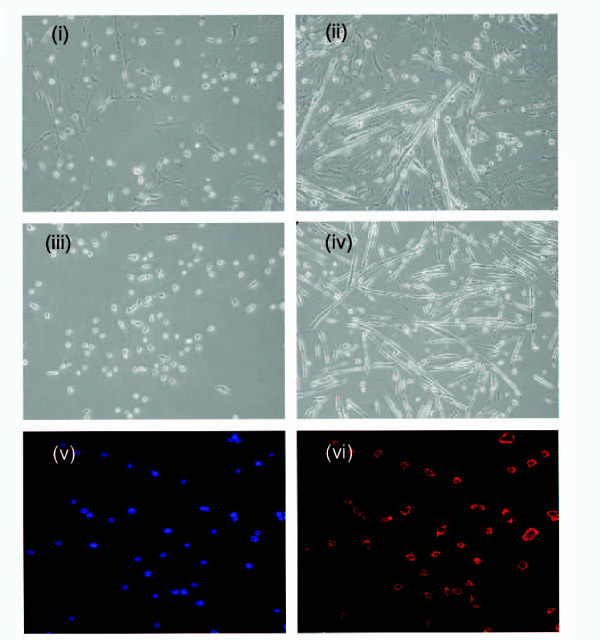
**Dystrophin repair in purified cultures of myoblasts**. Purification of the primary cultures of myoblasts. (i) enriched culture of myoblasts. The cultures were derived from limbs of Mdx5cv mice, and population of the myoblasts was enriched using pre-plating technique (see "Materials and Methods); (ii) myotubes in the enriched culture. The enriched cultures were induced to differentiation by culturing in differentiation medium. The arrows show representative fibroblasts; (iii) purified culture of myoblasts. The enriched cultures were subjected to pre-plating repeatedly until fibroblast-like cells were not observed in cultures. The purified cultures were cultivated in F10-based medium; (iv) myotube in the purified culture. The cultures were induced to differentiation by culturing in differentiation medium; (v) and (vi) desmin staining of the purified culture. Cell nuclei were counter stained with DAPI (v; blue). All of the observed cells were positive for desmin staining (vi; red).

We and others have shown that a transient knockdown of the mismatch repair protein Msh2 in repair proficient cells can increase the overall level of gene repair [26,30]. Msh2 is thought to inhibit the repair reaction through its action as an anti-recombinase [31,32] by precluding the binding of the oligonucleotide. Therefore, by knocking down the levels of this protein, we might predict that the oligonucleotide will bind more stably to the target site with higher efficiency and facilitate oligonucleotide binding. In order to determine whether the repair reaction could be stimulated by knocking down Msh2 in primary cells, we treated our cultures enriched in myoblasts with increasing concentrations of RNAi and quantified the amount of wild-type dystrophin DNA appearing after incubation with the targeting oligonucleotide for 24 hours. The correction frequency was enhanced 2 and 3 fold at the 1 nM and 10 nM concentrations of the RNAi (Figure 5). The efficiency decreases at higher concentrations of RNAi presumably due to the elevated levels of RNAi toxicity often observed in treated cells [33]. The enriched cell cultures used above were purified further to obtain myoblasts. This was accomplished by plating the cells on a collagen coated dish in F10 media. Once confluency was reached, the myoblasts were detached and separated from fibroblasts (etc.); this procedure was carried our repeatedly until the population of cells in the pooled culture was predominantly purified myoblast. The steps in this procedure are illustrated in Figure 6. Panels i and ii display the enriched culture of cells (i) and after differentiation into myotubes (ii); the arrows point to fibroblasts. In panels (iii) and (iv), the purified myoblast culture is illustrated before (i) and after (ii) differentiation into myotubes. Panels (v) and (vi) display cells in dark field after staining with Desmin (red). The fact that the vast majority of cells are stained in panel (vi) indicated that differentiation into myotubes has occurred. Thus, the procedure has enabled the isolation of a purified culture of myoblasts that can be targeted with the oligonucleotide.

**Figure 7:**
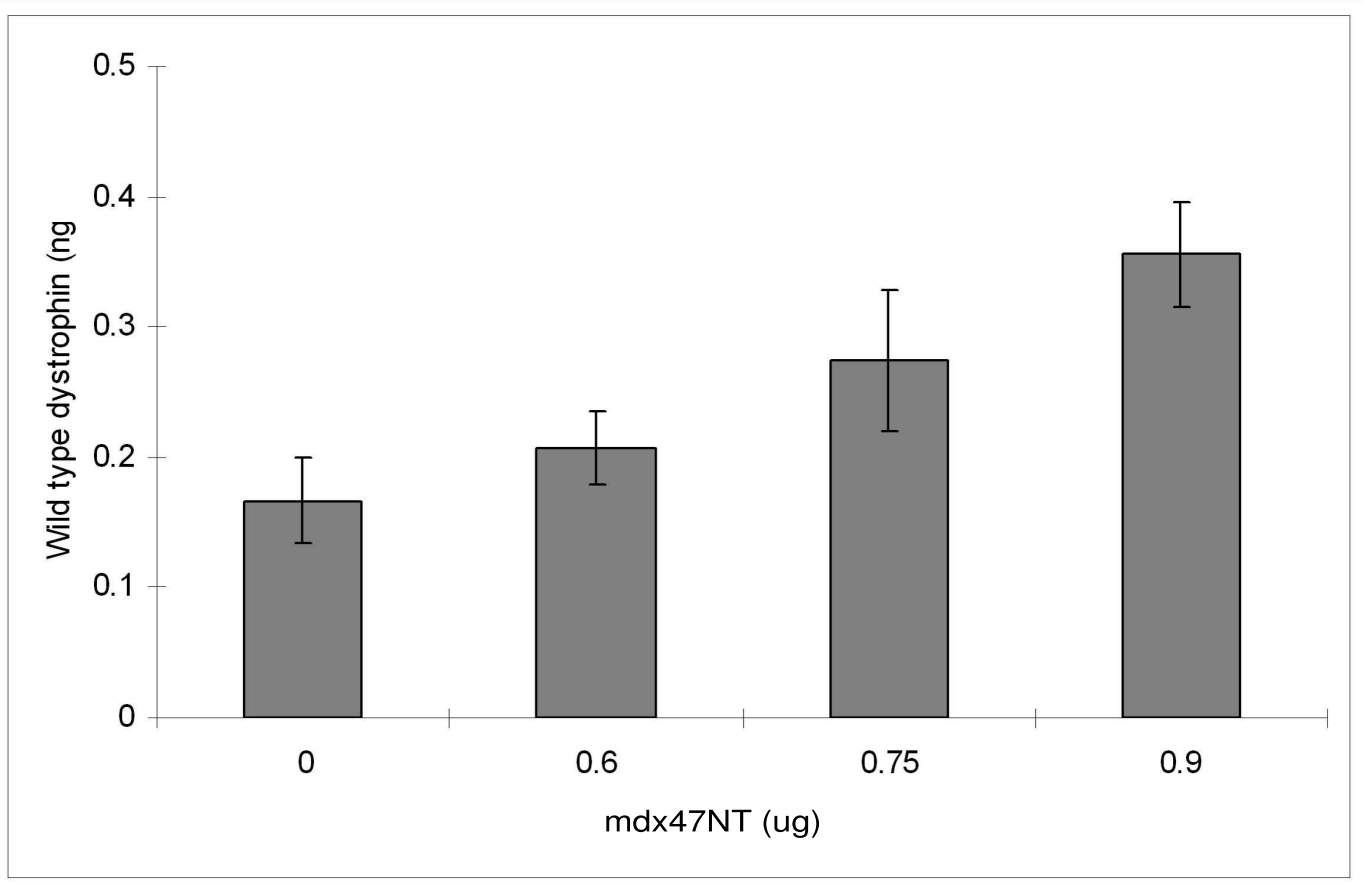
**Dystrophin correction in the purified cultures of myoblasts**. Various amounts of mdx47NT were transfected to purified myoblasts. Total genomic DNA was isolated after 24 hours, and 25 ng of this DNA was subjected to absolute quantification using a FAM labeled probe specific only for wild type dystrophin.

The mdx47NT ODN was introduced using jetPEI into a purified myoblast culture at various doses (the N/P ratio was kept at 5) and incubated for 24 hours. Genomic DNA was isolated and RT-PCR performed to analyze the samples for gene repair. We observed a clear dose response by calculating the amount of wild-type DNA emerging in treated samples (Figure 7) as compared to a standard curve (see Methods and Materials). The data suggest that a wild type dystrophin allele is present in at least some of the myoblasts where gene repair should have taken place. While it is not a perfectly sound comparison, the levels of gene repair in the enriched cultures and the purified cultures are approximately equivalent at the 24 hour time point (mdx47NT). Interestingly, the background level from the untreated control samples is higher in the purified myoblasts than in the enriched culture.

**Figure 8:**
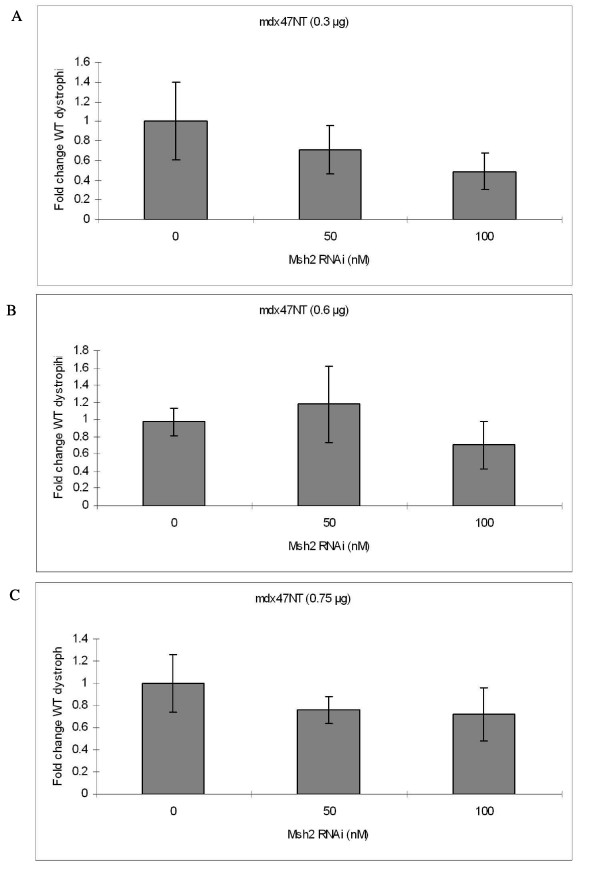
**Effect of Msh2 knockdown on dystrophin correction in purified myoblasts**. Purified myoblasts were co-transfected with various amounts of RNAi specific to knockdown Msh2 and (A) 0.3 μg, (B) 0.6 μg or (C) 0.9 μg of correcting oligonucleotide (mdx47NT), respectively. Total genomic DNA was isolated after 24 hours and 25 ng were subjected to absolute quantification using a FAM labeled probe specific only for wild type dystrophin. The fold change in wild-type dystrophin was determined from three independent experiments assayed in triplicate.

**Figure 9:**
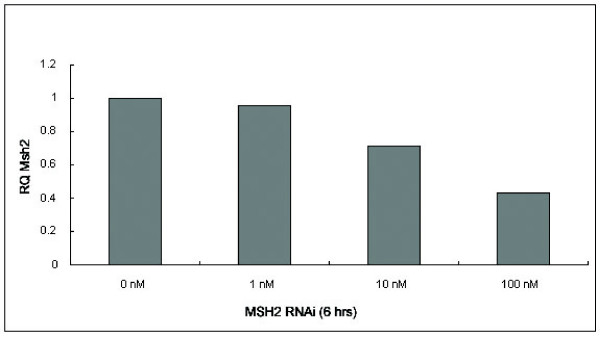
**Knockdown of Msh2 by RNAi**. Purified myoblasts were co-transfected with various amounts of RNAi specific to knockdown Msh2 and 0.75 μg of correcting oligonucleotide, mdx47NT. Total mRNA was isolated after 6 hours and subjected to RT-PCR.

Since RNAi-directed Msh2 knockdowns had elevated correction efficiencies in myoblast-enriched cultures, we applied this strategy to cultures of purified myoblasts where active conversion was shown (above) to take place. Purified myoblast cultures were treated simultaneously with various doses of the RNAi used in the experiments presented in Figure 5 and either 0.3 μg, 0.6 μg or 0.9 μg of targeting ODN, mdx47NT. The fold change in the evolution of wild type dystrophin DNA was measured after 24 hours and the results are presented in Figure 8A, B and 8C, respectively. No statistically significant difference in correction activity is observed at any RNAi level, although a clear reduction in Msh2 RNA levels is taking place (Figure 9). These data differ from the results reported in Figure 5 suggesting perhaps that enhancement of gene repair by RNAi may not have been occurring in myoblasts.

## Discussion

Successful correction of the dystrophin gene using either the RNA/DNA chimera or the modified single stranded oligonucleotide has been achieved in both dogs and mice by direct injection of synthetic DNA molecules [10-12,25]. The frequency of correction *throughout *muscle tissues however must be increased to enable therapeutic benefit. Bertoni *et al*. [25] have demonstrated that when muscle is injected directly with synthetic oligonucleotides, genetic correction was restricted to the dystrophin gene within a population of satellite cells. Since the frequency of gene repair is higher when delivery to cells outside the body takes place in an *ex vivo *approach. Thus, a more efficient approach for the treatment of DMD might entail using *ex vivo *delivery with subsequent transplantation back into the host.

To enable this *ex vivo *strategy, it was essential to optimize the efficiency of ODN delivery and ODN-mediated correction of myoblasts. This was accomplished by transfecting a culture of primary muscle cells enriched in myoblasts with a FAM labeled oligonucleotide and measuring uptake by FACs analysis. We confirmed that the ODN was present in the nucleus by examining the nuclei with confocal imaging (data not shown). Correction of the point mutation in the dystrophin gene was then demonstrated at the level of DNA, RNA and protein. Analysis of the quantity of corrected DNA indicated that the correction frequency remains stable over the course of 96 hours when targeting with either mdx47NT or mdx47T. A significant increase in message containing the portion of exon 10 deleted in the mutant cells was also detected at both 48 and 72 hours after transfection. There was no reduction in the level of repaired RNA over time which suggests that the corrected gene produces stable and inheritable message.

DNA analyses indicate that correction occurs at a 3 fold higher frequency when the nontranscribed strand of the gene is targeted. One hypothesis for strand bias is that the movement of RNA polymerase would interrupt the pairing of the ODN and this would result in lowered correction efficiency [34]. The dystrophin gene, however, is expressed only in differentiated cells and therefore, ODN binding stability should not be disrupted by the movement of the RNA polymerase. Alternatively, the orientation of the gene in terms of the general direction of replication fork movement can influence the targeting frequency in that the lagging strand targets at a higher level than the leading strand [35]. Thus, the strand designated as nontranscribed may also be the lagging strand at the target site.

Previous experiments of Ferrara and Kmiec [36] show that the corrected cells reduce their cell division cycle after introduction of the oligonucleotide because the free DNA ends activate G2 checkpoint proteins. During this time, the uncorrected population is able to divide, which will, in effect, cause the number of corrected cells to be diluted in number. This results in a lower measure of correction efficiencies as division in the uncorrected population is not impaired. In primary myoblasts cultures, however, this does not seem to be the case as the whole population of cells continues to divide with the correction efficiency maintained. This observation suggests that the corrected cells do not suffer a growth disadvantage by this *ex vivo *protocol.

Duchenne Muscular Dystrophy (DMD) is caused by an absence of the protein dystrophin as indicated by the absence of immunostaining for this protein in most of the muscle fibers. We observed elevated levels of dystrophin in cells that were treated with specific ODNs designed to correct the mutated dystrophin gene and after introduction of the oligonucleotide, myotubes that are expressing a significant amount of dystrophin are detected at 72 hours. The number of corrected cells that contribute dystrophin to the positive myotubes is unknown, but it is interesting to note that each myotube that stained positive had dystrophin evenly distributed throughout. This indicates that the dystrophin produced by the corrected gene can diffuse throughout a small myotube in vitro and would probably diffuse though part of a myofiber *in vivo*, in a region called the nuclear domain [37]. The long-term goal of the present study is to transplant corrected myoblasts back into mice to restore dystrophin production in muscle fibers. To achieve this goal it was necessary to establish *ex vivo *targeting conditions wherein the highest level of correction frequency in purified cells is achieved. The observation that Msh2 knockdown enhanced the repair frequency also indicates that the mechanism of gene repair is conserved in primary cells and has, to some degree validated basic studies in established model systems [38]. To our surprise, RNAi knockdown of Msh2 in cultures of purified myoblasts did not lead to an elevation in the repair of the mutant dystrophin gene, regardless of increasing amounts of correcting ODN at the varying doses of RNAi. The overall level of correction in the purified cultures is substantially lower and may be at a basal level where adjuvant treatments designed to stimulate activity do not impact the gene repair reaction. In addition, these results suggest that the stimulation of correction in the enriched cultures was likely taking place in the fibroblasts which contaminate the culture. For unknown reasons, primitive progenitor cells are less amenable to gene repair or at least provide an environment, less conducive for the reaction.

Correction levels vary among cell types with certain mutant targets being repair at levels approaching 1–5%. Yet, the consensus view is that progenitor cells, at any step along the differentiation pathway, do not enable this reaction to take place efficiently [39-41]. A series of natural barriers seems to exist in these cell types including lower transfection efficiencies and dosage-dependent toxicity. Increasing the amount of ODN introduced into the cell usually results in a gradual increase in side effects [39]. Progenitor cells are usually more sensitive to the transfection agent [42] and strand bias for targeting can also vary widely based in gene types [43]. Murphy *et al*. [39] found a mismatch hierarchy that contributed to the various levels of gene correction; A:G > A:A > A:C. If this is true, then the capacity to target progenitor cells will be hampered. Such a hierarchy has not been observed in somatic cell targeting where correction efficiencies are generally higher.

## Conclusion

Thus, progenitor cells will present unique challenges that will require exogenous manipulation to elevate frequency of gene repair. While MSH2 knockdown appears not to be a sufficient protocol to meet this goal in myoblasts, it does not mean that other progenitor lineages will not response positively. In fact, Dekker *et al*. [41] have already shown that MSH2 knockdown does increase the frequency of gene repair in mouse ES cells. More recently, Aarts *et al*. [30] provided support and confirmed these observation and Maguire and Kmiec [26] extended this protocol successfully into mouse embryonic fibroblasts. Morozov and Wowrousek [44] followed among the same theme with observations that inhibition of Rad51/54 and Ku70/86 led to an increase in gene correction efficiency. These types of cellular manipulations are certainly feasible and may be required for the successful application of gene repair in primary or progenitor cells.

## Methods

### Cells and oligonucleotides

Primary myoblasts derived from the mdx^5cv ^mice were maintained in DMEM supplemented with either 10% or 20% FBS. When the cells were induced to differentiate, DMEM containing 2% horse serum (DM) was added to the cells for at least 96 hours. The mutation of the parental plasmid pEGFP N3 to create the mutant pEGFP N3 (rep) has been described previously [38]. Targeting oligonucleotides, eGFP3S/47NT, mdx47NT, mdx47T, and mdx47NT-FAM were obtained either from Sigma Genosys (St. Louis, MO) or IDT (Coralville, IA) where they were synthesized and purified by HPLC before distribution. All modified single-stranded oligonucleotides were resuspended to a concentration of 200 μM and aliquots were stored at -20°C. The *mdx*^5*cv *^mice were obtained from the Jackson Laboratory.

### Isolation of myoblasts from newborn mdx^5cv ^mice

The isolation procedure for mouse myoblasts and subsequent pre-plating methods have been described previously [27,28]. This work was authorized by the Laval University Animal Care Committee and experiments were conducted in accordance with guidelines set forth by the Canadian Council of Animal Care. Briefly, limbs of 1 to 3 days old male *mdx*^5*cv *^mice were isolated and muscle was pulled from the bone and cartilage using sterile forceps. The muscle was treated for 1 hour with Collagenase (Sigma, St. Louis, MO) and Dispase II (Roche, Indianapolis, IN) to degrade collagen and to disrupt cell contacts. The cells were plated for 2 hours to allow fibroblasts, which rapidly adhere, to attach to the petri dish (PP2). The supernatant, containing a mixture of myoblasts and fibroblasts, was removed and the cells are passaged again to another petri dish (PP3). After 24 hours the cells were again passaged (PP4) and subsequently transferred to another culture dish (PP5). Purified myoblasts were expanded and used in gene repair experiments.

### Immunocytochemistry

Cells were aliquoted into 4 wells of an 8 well chamber at a density of 1 × 10^5 ^cells, and allowed to attach overnight. The cells were fixed using 100% ethanol for 5 minutes at -20°C, permeabilized with 0.1% Triton X-100, and then incubated with either desmin (Sigma, St. Louis, MO) specific or dystrophin specific antibodies raised in rabbit (Abcam, Cambridge, MA 1:1,000) at room temperature (RT) for 1 hour or overnight at 4°C. After washing the cells, a secondary goat α-rabbit antibody conjugated to a Cy3 fluorophore (desmin) (Zymed Laboratories, San Francisco, CA) or an Alexa 546-coupled goat anti-rabbit antibody (Molecular probes 1:1500) was added to the wells and this mixture was incubated with agitation for 1 hour at room temperature. After four 15 minute washes, the wells were dried and mounting media containing DAPI was added to each well (Molecular Probes, Eugene, OR). The cells were then visualized using a Zeiss™ inverted 100 M Axioscope equipped with a Zeiss™ 510 LSM confocal microscope with a coherent krypton argon laser (30 mW) containing 488 nm or 568 nm and 742 nm excitation lines.

### Gene repair of dystrophin in purified myoblasts

Cells were plated in 6-well culture dishes at a density of 1 × 10^5 ^with media containing 20% FBS. jetPEI was used to make oligonucleotide complexes with 3 μg of ODN at an optimal *N/P ratio *(nitrogen/phosphate ratio) of 5. This reagent was added to 1 ml of media and the cells were incubated for various amounts of time at which point differentiation media (DM) was added; the cells were differentiated for 96 hours, then harvested for FACS analysis and genomic DNA isolation. For mRNA analyses, the cells were collected at least four days after the addition of DM containing 2% horse serum.

### Knockdown of Msh2

RNAi, designed to knock down Msh2, was purchased from Ambion (Ambion, Austin, Texas); siRNA (ID# 155431) is designed to anneal to the mouse MSH2 RNA at exon 3 NM_008628. During the targeting experiments, the RNAi was mixed with the correcting oligonucleotides complexed with jetPEI and added to the cells as described above.

### Co-transfection ssODNs and Msh2 RNAi

Cells were plated in 6-well plates at a density of 1 × 10^5 ^cells/well with growth media (DMEM supplemented with 20% FBS, penicillin and streptomycin). Twenty four hours after plating, cells were co-transfected 3 μg of mdx/47NT and RNAi specific to knockdown Msh2 (0–200 nM). Seventy two hours after transfection, the growth media were replaced by differentiation media (DMEM supplemented with 2% horse serum, penicillin and streptomycin), and the cells were maintained in differentiation media for 96 h. Total RNA was isolated from the cells, and reverse transcription and quantitative PCR were conducted as described in "RNA isolation and, RT PCR and quantitative PCR.

### Msh2 RNAi

Cells were plated in 6-well plates at a density of 1 × 10^5 ^cells/well with growth media. Twenty four hours after plating, cells were co-transfected 3 μg of mdx47NT and RNAi specific to knockdown Msh2 (1–100 nM). Total RNA was isolated 3 or 6 h after transfection Methods of reverse transcription and quantitative PCR were same as "RNA isolation and, RT PCR and quantitative PCR", except for primers: musMSH2/257F 5' agaccctgcagagtgttgtgctta 3'; musMSH2/1010R 5' tgagagccagtggtgtcttcaaca 3' or mouse GAPDH4FwQ 5' tgtctcctgcgacttcaacagc 3'; GAPDH5RevQ 5' atgtaggccatgaggtccacca 3'.

### RNA isolation, RT PCR and quantitative PCR

After differentiation of the myoblasts, the cells were harvested by trypsinization and collected by centrifugation at 1,500 rpm for 7 minutes. After washing with 1× PBS, the RNA was extracted using a Qiagen RNA extraction kit (Qiagen, Valencia, CA) according to the manufacturer's instructions. Three hundred nanograms of RNA was subjected to reverse transcription to generate cDNA using a gene specific primer Rev-ex12 5' cag gtc caa agg gct ctt cc 3' or primer oligo dT using the first strand synthesis kit (Invitrogen, Carlsbad, CA). Five microliters was added to a 20 μL mixture of 2× Sybrgreen Master Mix (Applied Biosystems, Foster City, CA) with 2.5 μM of each primer:

Forw-ex 9 5' gtt atg cct tca cag gct gc 3'; Rev-ex10 in 5'cacttc ttc aac atc att tg3' or GAPDH4FwQ 5'tgtctcctgcgacttcaacagc3'; GAPDH5RevQ 5'atgtaggccatgaggtccacca 3' CKM F1 5' GCAACACCCACAACAAGTTCA 3' and CKM R1 5' TGTCTCCTTATCGCGAAGCTT 3'. Samples were added to each well of a 96 well plate in duplicate for each primer set. Dystrophin was amplified using the cDNA generated by the gene specific primer and GAPDH or CKM from cDNA generated using oligo dT. QPCR was run for 1 repetition at 50°C for 2 minutes, 1 repetition at 95°C for 10 minutes, 40 repetitions at 95°C for 15 seconds and 40 repetitions at 59°C for 1 minute with data collection at step 1 of stage 3. Upon completion of PCR, the threshold and baseline values were set to measure Ct values at the beginning of the exponential amplification. Ct values from 3 experiments were averaged per sample and subjected to a student's t-test and ANOVA to determine significance.

### DNA isolation and absolute quantification by PCR

Cells were harvested and collected by centrifugation at 1500 rpm for 7 minutes and washed twice with 1× PBS or lysed directly in a tissue culture dish after washing with 1× PBS. DNA was precipitated using the genomic wizard DNA extraction kit as instructed by the manufacturer (Promega, Madison WI). Twenty-five nanograms of genomic DNA was added to a total of 7 μL of water and this was mixed with 13 μL of a 2× PCR master mix, 50 nM of a 6FAM labeled probe specific to bind to wild-type dystrophin 5'FAMcac aag gag aga ttt3', 900 nM of forward primer mdx5cvasF 5' ttt ctg ccg agg ata cat tgc3' and 900 nM of reverse primer ex 10 out 5' ctc atg agc atg aaa ctg ttc 3'. Each experimental protocol was run in triplicate. A standard curve was generated using 50 ng, 25 ng, 12.5 ng, 6.25 ng, and 3.13 ng of genomic DNA isolated from C2C12 cells, which are wild-type for dystrophin. As a separate control, reaction mixtures containing 25 ng of genomic DNA from mdx^5cv ^were used to determine the background of wild-type DNA in each experiment. PCR conditions were: 1 repetition at 50°C for 2 minutes, 1 repetition at 95°C for 10 minutes and 40 cycles of 95°C for 15 seconds and 60°C for 1 minute. The data were collected at stage 3 of step 1. Threshold and baseline levels were set to measure the Ct values at the lowest level of exponential amplification and the average mean was determined from each experiment. Statistical significance was determined by a student's t-test and was considered significant only if *p *was less than 0.05. Following the PCR amplification the products were purified using Qiagen's QIAquick PCR purification kit according to the instructions and submitted for sequencing using the mdx5cvasF primer.

### Primary mouse myoblast purification

The enriched culture obtained from Tremblay lab was plated on a collagen-coated culture dish with F10 nutrient mixture (Invitrogen, Carlsbad, CA) supplemented with 10% (v/v) FBS, 2.5 ng/ml bFGF (Promega, Madison, WI), 100 U/ml penicillin and 100 mg/ml streptomycin. When the culture reached confluency, cells were detached from the dish without using trypsin. To accomplish this, the dish was washed with PBS after aspiration of medium, incubated with a small amount of PBS at room temperature for 5 min, and hit firmly sideway**s**. Predominant cell type in the detached cells was myoblast due to the fact that myoblasts are likely to detach from collagen-coated dish than fibroblasts are. The detached cells were collected with F10 nutrient mixture, plated on a new collagen-coated dish, and expanded. The above procedures were repeated until no fibroblasts were observed in the culture. Upon completion of purification, medium was switched F10 growth medium to F10/DMEM (1:1) supplemented with 10% (v/v) FBS, 2.5 ng/ml bFGF (Promega, Madison, WI), 100 U/ml penicillin and 100 mg/ml streptomycin.

### Desmin staining

Cells were plated on 2-well chambers at a density of 8 × 10^4 ^cells/well and incubated at 37°C overnight. The cells were fixed using ethanol for 5 minutes at -20°C, permeabilized using 0.3% (v/v) TritonX for 10 minutes at room temperature, blocked using 5% (v/v) heat-inactivated goat serum for 30 minutes at room temperature, and then incubated with desmin specific antibody (Sigma, St. Louis, MO; dilution, 1:50) for 1 hour at room temperature. After washing the cells, a secondary goat a-rabbit antibody conjugated to Cy3 (Zymed laboratories, San Francisco, CA; dilution, 1:750) was added to the wells and this incubated with agitation for 1 hour at room temperature. After four 15 minute washes, the wells were briefly dried and mounting media containing DAPI was added to each well (Molecular Probes, Eugene, OR).

### Oligo dose experiment

Purified myoblasts were plated in 6-well plates at a density of 1.5 × 10^5 ^cells/well with F10/DMEM growth medium 24 hours before transfection. jetPEI was used to make oligonucletide complexes with various amounts of mdx47NT at N/P (nitrogen/phosphate) ratio of 5. This reagent was added to 1 ml of media and the cells were incubated. Genomic DNA was isolated 24 hours after transfection using masterpure DNA purification kit (Epicentre, Madison, WI). TaqMan^® ^probe-based real time PCR was performed to quantify wild type dystrophin. Probe specific for wild type dystrophin (5'-FAMcac aag gag aga ttt-3') were used with the primer pair: forward primer, 5'-ttt ctg ccg agg ata cat tgc-3'; reverse primer, 5'-ctc atg agc atg aaa ctg ttc-3'. Real time PCR was performed with 25 ng genomic DNA, 900 nM both forward and reverse primer, and 250 nM probe in each 20 ml reaction. A standard curve was generated using 1, 0.5, 0.25, 0.125, 0.0625 ng of genomic DNA isolated from C2C12 cells, which have wild type dystrophin gene. PCR conditions were: 1 repetition at 50°C for 2 minutes, 1 repetition at 95°C for 10 minutes and 40 cycles of 95°C for 15 seconds and 60°C for 1 minute. Each experimental protocol was run in triplicate.

### Co-transfection ssODNs and Msh2 RNAi

Purified myoblasts were plated in 6-well plates at a density of 1.5 × 10^5 ^cells/well with F10/DMEM growth medium 24 hours before transfection. Cells were co-transfected 0.75 mg of mdx47NT and siRNA for Msh2 knockdown (0–100 nM) using jetPEI. Twenty four hours after transfection, genomic DNA was isolated, and TaqMan^® ^probe-based real time PCR was performed to quantify wild type dystrophin as described above ("Oligo dose experiment")

### Msh2 RNAi

Purified myoblasts were plated in 6-well plates at a density of 1.5 × 10^5 ^cells/well with F10/DMEM growth medium 24 hours before transfection. Cells were co-transfected 0.75 mg of mdx47NT and siRNA for Msh2 knockdown (0–100 nM) using jetPEI. Total RNA was isolated 6 hours after transfection. Two hundred nanograms of RNA was subjected to reverse transcription to generate cDNA using the first strand synthesis kit (Invitrogen). Five microliters was added to a 20 ml mixture of 2× Sybergreen master mix (Applied Biosystems, Foster City, CA) with 2.5 mM of each primer: musMSH2/257F 5' agaccctgcagagtgttgtgctta 3'; musMSH2/1010R 5' tgagagccagtggtgtcttcaaca 3' or mouse GAPDH4FwQ 5' tgtctcctgcgacttcaacagc 3'; GAPDH5RevQ 5' atgtaggccatgaggtccacca 3'. Samples were added to each well of a 96 well plate in duplicate for each primer set. QPCR was run for 1 repetition at 50°C for 2 minutes, 1 repetition at 95°C for 10 minutes, 40 repetitions at 95°C for 15 seconds and 40 repetitions at 59°C for 1 minute with data collection at step 1 of stage 3. Upon completion of PCR, the threshold and baseline values were set to measure Ct values at the beginning of the exponential amplification. Ct values from 3 experiments were averaged per sample.

## Abbreviations

ODN: Single-stranded oligonucleotides; DMD: Duchenne Muscular Dystrophy.

## Authors' contributions

KM carried out the experiments in enriched myoblasts along with the initial RNAi work; TZ purified the myoblasts and carried out the experiments in the purified cells. KM and HPO in conjunction with EK conceived the study; HPO and EK drafted the manuscript. All authors read and approved the final manuscript.
